# A Review of Strategies to Improve the Electrocatalytic Performance of Tungsten Oxide Nanostructures for the Hydrogen Evolution Reaction

**DOI:** 10.3390/nano15151163

**Published:** 2025-07-28

**Authors:** Meng Ding, Yuan Qin, Weixiao Ji, Yafang Zhang, Gang Zhao

**Affiliations:** School of Physics and Technology, University of Jinan, 336 Nanxinzhuang West Road, Jinan 250022, China; qy975468@163.com (Y.Q.); sps_jiwx@ujn.edu.cn (W.J.); sps_zhangyf@ujn.edu.cn (Y.Z.); sps_zhaog@ujn.edu.cn (G.Z.)

**Keywords:** tungsten oxide, hydrogen evolution reaction, electrocatalytic activity, optimizing performance

## Abstract

Hydrogen, as a renewable and clean energy with a high energy density, is of great significance to the realization of carbon neutrality. In recent years, extensive research has been conducted on the electrocatalytic hydrogen evolution reaction (HER) by splitting water, with a focus on developing efficient electrocatalysts that can perform the HER at an overpotential with minimal power consumption. Tungsten oxide (WO_3_), a non-noble-metal-based material, has great potential in hydrogen evolution due to its excellent redox capability, low cost, and high stability. However, it cannot meet practical needs because of its poor electrical conductivity and the limited number of active sites; thus, it is necessary to further improve HER performance. In this review, recent advances related to WO3-based electrocatalysts for the HER are introduced. Most importantly, several tactics for optimizing the electrocatalytic HER activity of WO_3_ are summarized, such as controlling its morphology, phase transition, defect engineering (anion vacancies, cation doping, and interstitial atoms), constructing a heterostructure, and the microenvironment effect. This review can provide insight into the development of novel catalysts with high activity for the HER and other renewable energy applications.

## 1. Introduction

With the increasing severity of the energy crisis and environmental problems, the world has reached a consensus on the goal of “carbon neutrality” and taken action. Therefore, it is urgent to develop green, safe, and renewable energy as an alternative to traditional fossil fuels [1,2,3]. Hydrogen (H_2_), which has a high energy density (about 120 kJ/g) and is environmentally friendly [4], will play a remarkable role in the renewable energy system in the future and will be conducive to achieving the goals of carbon dioxide emission reductions and carbon neutrality [5,6]. At present, hydrogen can be produced by coal gasification [7], methane cracking [8], and reforming [9] methods using fossil fuels such as natural gas, coal, and oil as raw materials. Nearly 96% of the world’s hydrogen is produced by the conversion of conventional fossil fuels. These processes are accompanied by the production of large amounts of CO_2_, sulfur oxides, and other waste gases, and their energy consumption is large, which could aggravate the global energy crisis and environmental pollution. However, the production of “green hydrogen” using renewable energy sources integrated with water electrolysis can achieve zero carbon emissions and is essential for a carbon-neutral strategy [6].

The water electrolysis reaction generally consists of two half-reactions: the hydrogen evolution reaction (HER) and the oxygen evolution reaction (OER) [10]. The catalyst plays a crucial role in the electrocatalytic reaction, and a highly efficient electrocatalyst can significantly lower the energy consumption required for the reaction. Currently, electrocatalysts depend primarily on precious-metal-based materials, including Pt-based HER electrocatalysts [11,12] and Ir/Ru-based OER electrocatalysts [13,14,15,16,17]. However, the high costs and scarcity of precious-metal-based electrocatalytic materials have hindered their widespread application [18,19]. Therefore, researchers have devoted themselves to the development of sustainable non-precious-metal electrocatalytic materials with low costs and high efficiency and stability. At present, great progress has been made in research on transition metal-based electrocatalysts, including oxides [20,21,22], hydroxide/hydroxyl oxides [23,24,25,26], sulfides [10,25,27,28,29,30], phosphide [31,32,33], selenide [34,35], nitrides [36,37], and carbides [38,39,40], which are much better alternatives to precious-metal catalysts due to their abundant reserves, low prices, environmental friendliness, and great potential for improving catalytic performance.

At present, transition metal oxides (TMOs), including Fe, Co, Ni, W, and Mn oxides, are attracting attention as potential electrocatalytic materials [41,42,43,44,45]. In contrast to the common precious-metal catalysts, transition metal oxides have the advantages of lower costs and easier accessibility, making them more feasible for commercial applications [46]. Tungsten has a great number of stable oxidation states, so it has a variety of electrochemical properties; among these states, tungsten oxide is the most stable. Tungsten oxide is a typical n-type semiconductor with a band gap of 2.6–2.8 eV [47] and electron mobility of about 12 cm^2^·V^−1^·s^−1^ [48]. The crystal structure of WO_3_ belongs to the ReO_3_ type, which is similar to the perovskite structure of ABO_3_ except with the absence of the A-site cation. It can be regarded as having a W atom at the center surrounded by six oxygen atoms, with the basic unit being a regular octahedron formed by WO_6_. According to the rotation direction and tilt angle of the WO_6_ octahedron, the crystalline phase of WO_3_ can be divided into monoclinic II (ε-WO_3_), triclinic (δ-WO_3_), monoclinic I (γ-WO_3_), orthorhombic (β-WO_3_), tetragonal (α-WO_3_), and cubic WO_3_ [49], which are widely used in the fields of energy storage [50], sensors [51,52], and catalysis [53,54,55], among others. WO_3_, as a transition metal oxide, is a promising electrocatalyst for an efficient HER because of its excellent redox capability, low cost, and high stability [56]. However, its electrical conductivity is poor, and active sites are limited, which result in the poor HER performance of WO_3_. The conductivity of non-metrological WO_3−x_, with oxygen vacancies and defect structures, such as WO_2_ [57,58], WO_2.72_ [59,60], WO_2.83_ [61], WO_2.9_ [20], and WO_2.92_ [62], is significantly improved. Of course, a lot of effort has been made in regulating and activating WO_3−x_ to improve their HER performance.

Based on previously reported research results, this review first summarizes recent advances in stoichiometric WO_3_ for the electrocatalytic HER. Then, this review chiefly discusses recent advances in different strategies for improving the catalytic activity of WO_3_-based materials, such as controlling their morphology, defect engineering, interfacial engineering, tailoring the microenvironment, and so on. Finally, the main conclusions and perspectives related to HER electrocatalysts are discussed. It is hoped that this review can bring some inspiration and reference value to future research on WO_3_ or other related catalysts.

## 2. WO_3_ Nanostructures for HER

The stoichiometric WO_3_ nanostructures used as electrocatalysts for the HER include nanoparticles, nanorods, nanowires, nanoplates, and so on. WO_3_ nanostructures have been synthesized using hydrothermal [63,64], sonochemical [65], calcining [66], ionic exchange [67], inorganic–organic hybrid [68], and ultrasonic processes [69], among others. Due to the quantum size effect and their large specific surface areas, the physical and/or chemical properties of nanomaterials deviate from those in the bulk phase. For example, Ganesan et al. prepared WO_3_ nanoparticles by calcining with a chitosan biopolymer as a template. They exhibited fourfold higher catalytic activity than bulk WO_3_ for the HER in 1.0 M H_2_SO_4_ [66]. In addition, a nanostructure with a one-dimensional (1D) morphology (e.g., nanorods and nanowires) can provide an enlarged electrode–electrolyte interface to facilitate reaction kinetics while simultaneously enabling rapid electron transfer. Consequently, electrocatalysts with 1D architectures can exhibit superior electrocatalytic hydrogen evolution properties. For example, Rajeswari et al. [70] synthesized WO_3_ nanorods by pyrolysis of tetrabutylammonium decatungstate at 450 °C, and the WO_3_ nanorods showed much better HER catalytic performance compared with the bulk phase due to the unique electrochemical behavior of 1D nanostructures. Lee et al. [71] prepared hexagonal WO_3_ nanowires with a large surface area and a large pore volume using a microwave-assisted hydrothermal process. They exhibited excellent HER activity, which was ascribed to their high aspect ratio and crystallinity. Lee et al. [63] synthesized monoclinic WO_3_ (m-WO_3_) nanoplates and nanorods using a hydrothermal method, and the m-WO_3_ nanorods displayed slightly better electrocatalytic activity for the HER than the m-WO_3_ nanoplates. The experimental results further demonstrated that the 1D nanostructure is not only conducive to increasing the number of active sites for reaction but also enables rapid transfer of electrons along the crystal growth direction. Other studies researching the electrocatalytic hydrogen evolution of pure WO_3_ have been reported, as summarized in Table 1. It can be seen that the electrocatalytic HER performances of 1D nanostructures (e.g., nanorods and nanowires) and two-dimensional (2D) nanostructures (e.g., nanoplates and nanosheets) are comparable, and both significantly outperform that of nanoparticles. Additionally, their HER activity in acidic electrolytes surpasses that in alkaline electrolytes and is much better than that in neutral solutions.

Many significant research efforts have been devoted to studying WO_3_ electrocatalysts, achieving notable advancements in catalytic performance, while the intrinsic activity of WO_3_ nanostructures remains unsatisfactory. Consequently, further designing efficient WO_3_-based electrocatalysts is imperative for practical application of hydrogen energy.

## 3. Different Strategies for Improving Electrocatalytic Performance of WO_3_ for HER

The electrocatalytic HER activity of a material is fundamentally governed by critical parameters, including the charge transport capability, the accessibility of active sites, and electronic structure configurations [76]. Therefore, multifaceted strategies such as controlling the morphology [77], tailoring the phase [74], doping, constructing a heterostructure, and microenvironment modulation [64,78] have been proposed to systematically enhance the intrinsic activity and reaction kinetics of electrocatalysts.

### 3.1. Morphological Control

Electrocatalytic reactions primarily occur on the surfaces of catalysts, where specific morphologies (e.g., nanosheets, porous structures, and nanowires) are favorable for the exposure of highly active crystal planes or edge sites, thereby increasing the density of active sites. Additionally, nanostructures (such as three-dimensional (3D) porous and hierarchical architectures) can significantly increase the specific surface area to provide additional reaction interfaces, thereby simultaneously promoting electrolyte penetration and gas bubble detachment to reduce mass transport resistance. Unique configurations (e.g., core–shell structures and self-supporting electrodes) effectively suppress catalyst aggregation and dissolution during reactions, thereby prolonging the service life and improving the stability of electrocatalysts. Therefore, construction and preparation of electrocatalytic materials with specific microstructures is an effective strategy to optimize the behavior of catalysts.

**Figure 1:**
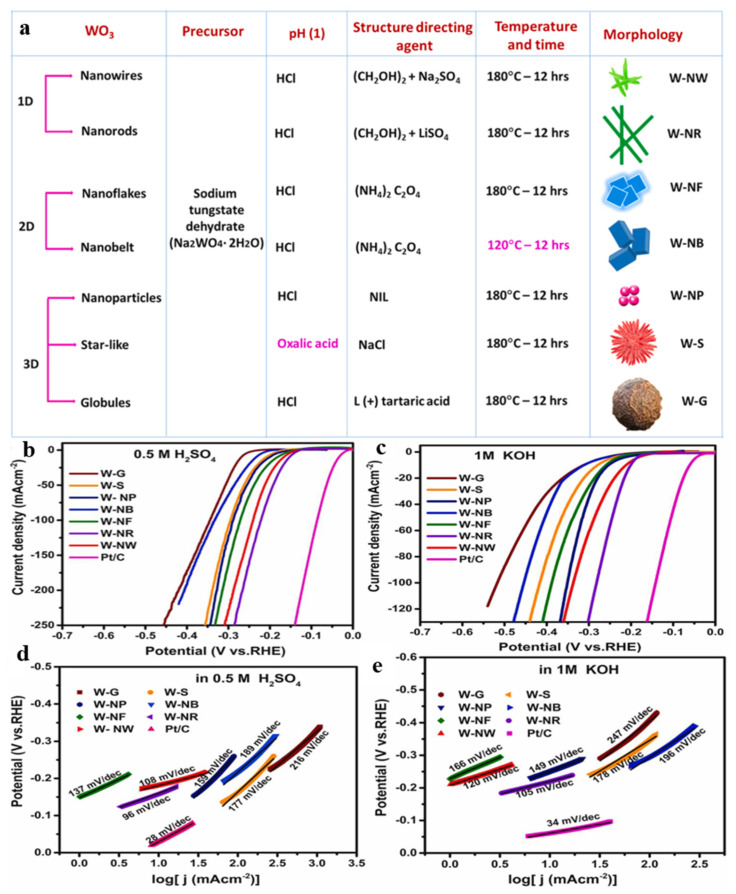
(**a**) Synthesis protocol for WO_3_ electrocatalysts with different morphologies. LSV curves of WO_3_ electrocatalysts in (**b**) 0.5 M H_2_SO_4_ and (**c**) 1 M KOH_._ Tafel plots of WO_3_ electrocatalysts in (**d**) 0.5 M H_2_SO_4_ and (**e**) 1 M KOH [74].

The morphologies and crystallographic phases of nanostructures can be controlled by synthetic techniques, conditions, and parameters. Lee et al. [63] prepared monoclinic-phase WO_3_ using hydrothermal synthesis by adding and tuning the amount of ammonium nitrate (NH_4_NO_3_). Furthermore, the morphology of m-WO_3_ was transformed from nanoplates to nanorods by adding polyethylene glycol in solution, making its HER activity much higher than that of commercial bulk m-WO_3._ Moreover, the catalytic activity of the m-WO_3_ nanorods was superior to the nanoplates in an acidic medium, which could be attributed to the increased active sites and improved electron transfer along the crystal growth direction in the unique 1D morphology. Zhang et al. [73] synthesized monoclinic hierarchical flower-like structures consisting of nanoplates and hexagonal nanorods of WO_3_ by adjusting the concentration of hydrochloric acid (HCL) used in the hydrothermal method. The hexagonal phase of WO_3_ composed of nanorods required a low overpotential of 55 mV to achieve a current density of 10 mA·cm^2^ and exhibited excellent electrochemical stability in an acidic medium. It demonstrated much better catalytic activity than the hierarchical monoclinic phase. Ponpandian et al. [74] adjusted the hydrothermal synthesis scheme according to surfactants and physical parameters (temperature and time) and synthesized WO_3_ electrocatalysts with different morphologies, including 1D-WO_3_ nanowires (W-NWs), WO_3_ nanorods (W-NRs), 2D-WO_3_ nanobelts (W-NBs), WO_3_ nanoflakes (W-NFs), 3D-WO_3_ nanoparticles (W-NPs), star-like WO_3_ (W-S), and WO_3_ globules (W-Gs), as displayed in Figure 1a. The linear sweep voltammetry (LSV) curves of the as-prepared WO_3_ electrocatalysts with different morphologies and Pt/C were investigated in acidic and alkaline solutions (Figure 1b,c), respectively. Obviously, the overpotentials of the samples with the same current density in acidic media were lower than that of the alkaline solution. In particular, the W-NRs displayed a much better electrocatalytic HER performance than the other catalysts, and their overpotentials were 152 and 201 mV at 10 mA·cm^−2^ in 0.5 M H_2_SO_4_ and 1 M KOH solutions, respectively. Furthermore, the Tafel slopes of the W-NR electrocatalyst (Figure 1d,e) were 96 mV·dec^−1^ and 105 mV·dec^−1^ in 0.5 M H_2_SO_4_ and 1 M KOH solutions, respectively. This work has a promising scope for constructing highly efficient interfacial active sites required for large-scale hydrogen production by tuning the morphology and crystalline structure of metal oxides at the molecular level.

### 3.2. Phase Transition

The intrinsic activity of catalysts fundamentally relies on their crystal structure and surface electronic state [76,79,80], and the latter could be adjusted by phase transitions [81]. Phase transitions can be induced by external stimuli including electric fields [82], pressure [83], temperature [84], and strain [85,86], which could optimize the intrinsic activity and quantity of active sites. Duerloo et al. discovered that application of stress to transition metal dichalcogenides (TMDs) can induce a phase transition from the 2H phase to the 1T phase [86]. Zhang et al. successfully fabricated curved and conformally wrapped cobalt-doped tungsten selenide (Co-WSe_2_) nanosheets on carbon nanotubes. This curved growth mode enhanced the anisotropy of the material, thereby boosting its intrinsic catalytic activity. Concurrently, the strain generated by the conformal coating further promoted the formation of the metallic 1T phase, significantly improving the electrical conductivity. The strain effects induced by small-diameter tubular multi-walled carbon nanotubes (MWNTs) endowed the catalyst with enhanced catalytic activity and long-term stability [85]. Zhou et al. demonstrated that phase transition from the polymorphic δ phase to the α phase can be induced in MnO_2_ by adjusting the hydrothermal time. Furthermore, experimental and theoretical density functional theory (DFT) calculation results showed that an α-MnO_2_ polymorph exhibited much better electrocatalytic activity than δ-MnO_2_ due to a compatible energy band gap and superior band alignment tunability [80].

**Figure 2:**
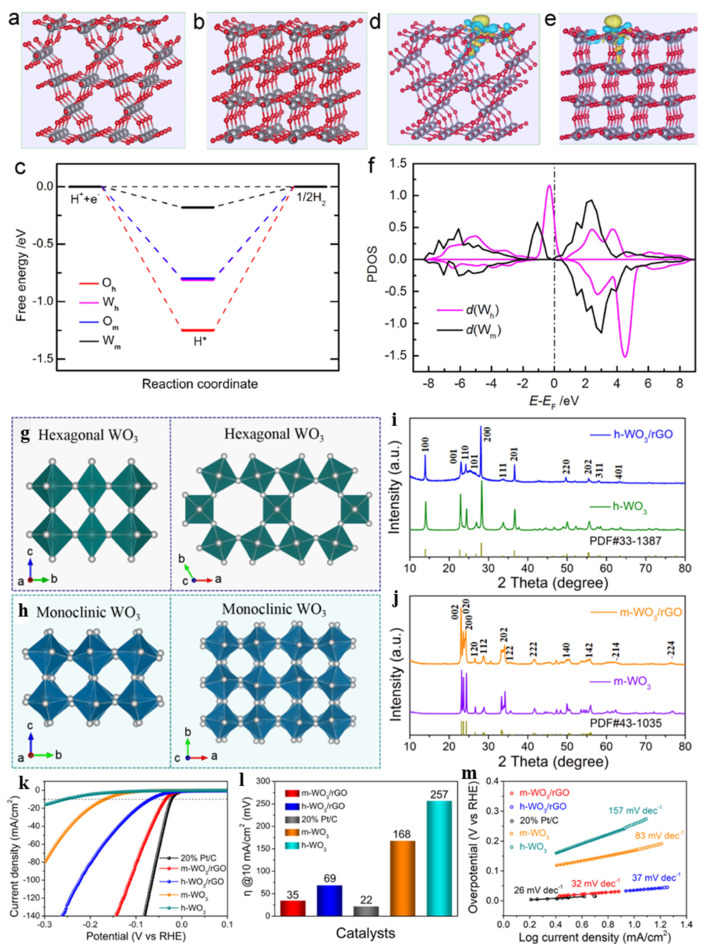
The optimized geometric configurations of (**a**) h-WO_3_ (100) and (**b**) m-WO_3_ (002) surfaces with W atoms (gray spheres) and O atoms (red spheres). (**c**) Calculated HER free energy profiles under equilibrium for W and O active sites on h-WO_3_ (100) and m-WO_3_ (002) surfaces. Three-dimensional contour plots of charge density differences for H adsorption at W sites on (**d**) h-WO_3_ (100) and (**e**) m-WO_3_ (002) surfaces. The yellow and light blue areas imply electron accumulation and depletion, respectively. (**f**) PDOS analysis of the d orbital for W atoms on h-WO_3_ (100) and m-WO_3_ (002) after hydrogen adsorption. Crystalline structure diagrams of the (**g**) hexagonal and (**h**) monoclinic phases. XRD of (**i**) h-WO_3_ and (**j**) m-WO_3_. (**k**) LSV curves. (**l**) The overpotentials required at 10 mA·cm^−2^ for different catalysts. (**m**) Tafel slopes of as-grown samples. All electrochemical measurements were performed in a 0.5 M H_2_SO_4_ solution at room temperature [88].

Considering the effect of the crystal structure on the electronic structure and adsorption energy of WO_3_, Ponpandian [74] prepared different crystal phases and morphologies of WO_3_ using the hydrothermal method. The solvent, surfactant, and synthesis parameters (notably the reaction temperature and time) synergistically determined the structural orientation of the nanostructures. Xie [87] et al. synthesized a series of Pt-WO_3_ catalysts using the hydrothermal method, and the phase transition from WO_3_ to H_x_WO_3_ caused by an electrochemical H^+^ insertion/removal process was verified by in situ Raman spectroscopy analysis in Pt-WO_3_. H_x_WO_3_ was found to facilitate electron transfer dynamics and hydrogen transport from H_x_WO_3_ to adjacent Pt sites during the HER. Yang et al. [88] have investigated the relationship between intrinsic activity and the crystal structure in the hexagonal and monoclinic phases of WO_3_ (h-WO_3_ and m-WO_3_) during the HER. The WO_2_ terminations for the h-WO_3_ (100) and m-WO_3_ (002) surfaces were selected for investigation, as shown in Figure 2a,b. The computed energy profiles of the reaction coordinates for hydrogen adsorption (Figure 2c) demonstrated that the strong O-H binding interaction suppresses H_2_ desorption while concurrently diminishing the catalytic activity of bridge O atoms. Electronic structure analysis was employed to elucidate the hydrogen adsorption abilities of the h-WO_3_ (100) and m-WO_3_ (002) surfaces, and the calculation results (Figure 2d,e) implied weaker W-H bonds and higher catalytic activity on m-WO_3_ (002). Furthermore, the d-orbital projected density of state (PDOS) of surface W atoms after H adsorption on the WO_3_ surface is plotted in Figure 2f. Comparative d-orbital PDOS analysis revealed that the h-WO_3_ (100) surface exhibits a prominent d-band across the Fermi level, in contrast to the m-WO_3_ (002) surface. This elevation of the d-band near the Fermi level in the electronic configuration significantly facilitated the formation of W-H bonds. The calculated results conclusively demonstrated that the lower H-adsorption energy and weaker W-H bonding on the m-WO_3_ (002) surface synergistically accelerated the desorption of surface-adsorbed H* intermediates. According to DFT calculations, the m-WO_3_ displayed a suitable energy barrier for the H-adsorption/desorption energy, which could be favorable for rapid desorption of active H* intermediates compared with h-WO_3_, resulting in excellent HER catalytic activity. The h-WO_3_ and m-WO_3_ were prepared by the hydrothermal method and calcined with different temperatures under a N_2_ atmosphere, and the XRD spectra of all samples are shown in Figure 2i,j. The HER was performed in a 0.5 M H_2_SO_4_ solution at room temperature, and the experimental results for m-WO_3_ and h-WO_3_ are shown in Figure 2k–m. The m-WO_3_ showed a low Tafel slope of 83 mV·dec^−1^, and an overpotential of 168 mV was required to realize a current density of 10 mA·cm^−2^. For the h-WO_3_, the overpotential was 257 mV at 10 mA·cm^−2^ and the Tafel slope was 157 mV·dec^−1^. Obviously, the m-WO_3_ exhibited much higher electrocatalytic activity than the h-WO_3._ Consequently, to attain highly active WO_3_ catalysts, maximizing monoclinic-phase WO_3_ is recommended in experiments.

### 3.3. Defect Engineering

Introducing intrinsic defects in transition metal-based electrocatalysts can generate a large number of active sites, increase conductivity, modulate electronic states, promote ion diffusion, and thus enhance catalytic performance. Therefore, studying the regulation of intrinsic defects in non-precious-metal electrocatalysts is of great importance to improve their energy conversion efficiency. The defects include edge defects, vacancy defects [89], and doping-derived defects [90]. Introducing lattice vacancy or atom doping can destroy the periodic arrangement of crystalline structures and can affect the local electronic environment to form unsaturated coordination states. Moreover, the hydrogen adsorption/dissociation energy can also be optimized by defect engineering [91,92].

#### 3.3.1. Anion Vacancies

**Figure 3:**
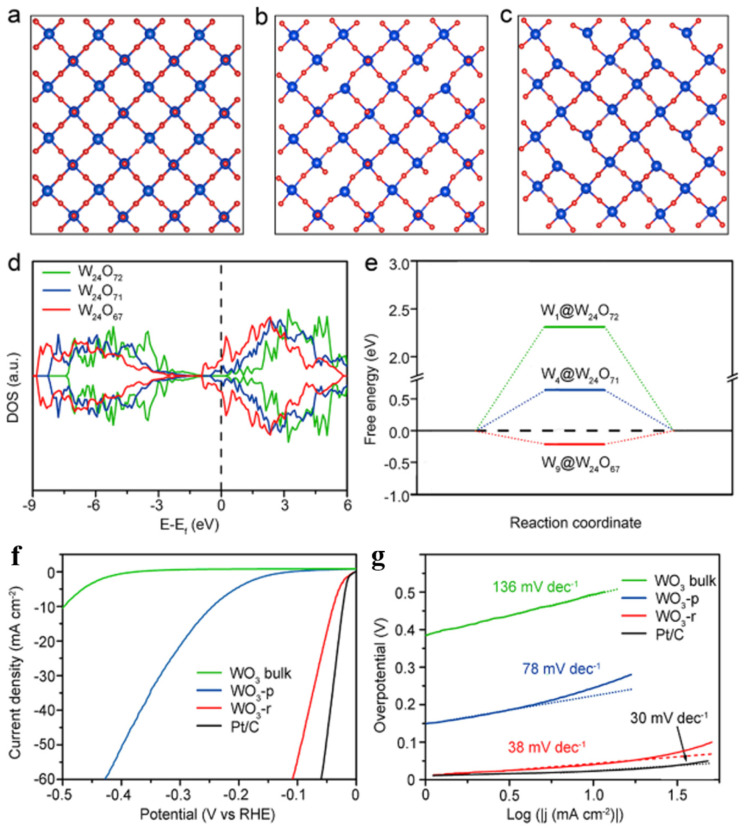
The crystal structures of WO_3_ (010) with (**a**) an ideal surface (labeled as W_24_O_72_), (**b**) a surface with one bridging V_O_ (labeled as W_24_O_71_), and (**c**) a surface with all terminal O atoms and one bridging O atom removed (labeled as W_24_O_67_). (**d**) The DOS of W 5d. (**e**) The ΔG_H_ at the W site on WO_3_ (010) with low and high concentrations of V_O_. (**f**) LSV curves. (**g**) Tafel plots of different samples measured in H_2_-saturated 0.5 M H_2_SO_4_ [89].

Anion vacancies are one of the most widespread intrinsic defects. For example, oxygen vacancies widely exist in transition metal oxides due to their low formation energy, which could change the physicochemical properties of oxides [45,93,94,95]. It has been reported that the oxygen vacancies in TMOs are crucial for efficient hydrogen evolution. Especially, introduction of oxygen vacancies usually leads to variation in the oxidation state of a metal, accompanied by the formation of new electron states in the band gap and an increase in the carrier concentration. Wu and their coworkers [93] prepared WO_2_–carbon mesoporous nanowires (MWCMNs) with a high concentration of oxygen vacancies (V_O_) by calcinating hybrid WO_3_ and ethylenediamine precursors, and they exhibited excellent HER activity of 58 at 10 mA·cm^−2^ and a Tafel slope of 46 mV·dec^−1^. Experimental and DFT calculation results confirmed that the presence of a large number of V_O_ played a vital role in the introduction of an unusual electron state near the Fermi level and provided more active sites in the MWCMNs. Zhou et al. [89] constructed a hydrogen adsorption model for WO_3_ and calculated the electronic structure of WO_3_ with oxygen vacancy using density functional theory. Three different structural models were built on the basis of a WO_3_ (010) (√2 × √2) R45° slab, as shown in Figure 3a–c. According to the density of state (DOS) results, introducing oxygen vacancies could make new energy levels for W-5d at the conduction band minimum (CBM). Furthermore, the increasing concentration of oxygen vacancies endowed the WO_3_ slab with a remarkably aggrandized DOS of W 5d at the CBM. The energy gap introduced by V_O_ could transform a metal oxide from a traditional semiconductor to a degenerate semiconductor with high conductivity. The variation in the free energy (ΔG_H_) decreased to close to zero, which promoted hydrogen adsorption (Figure 3e). In the experiment, WO_3_ nanosheets with rich V_O_ could be obtained by liquid exfoliation of the tungsten oxide precursors, and they exhibited superior HER activity (Figure 3f,g) with a low overpotential of 38 mV at 10 mA·cm^−2^ and a Tafel slope of 38 mV·dec^−1^. It can thus be seen that the experimental results were correspondent with the theoretical calculations.

#### 3.3.2. Cationic Doping

**Figure 4:**
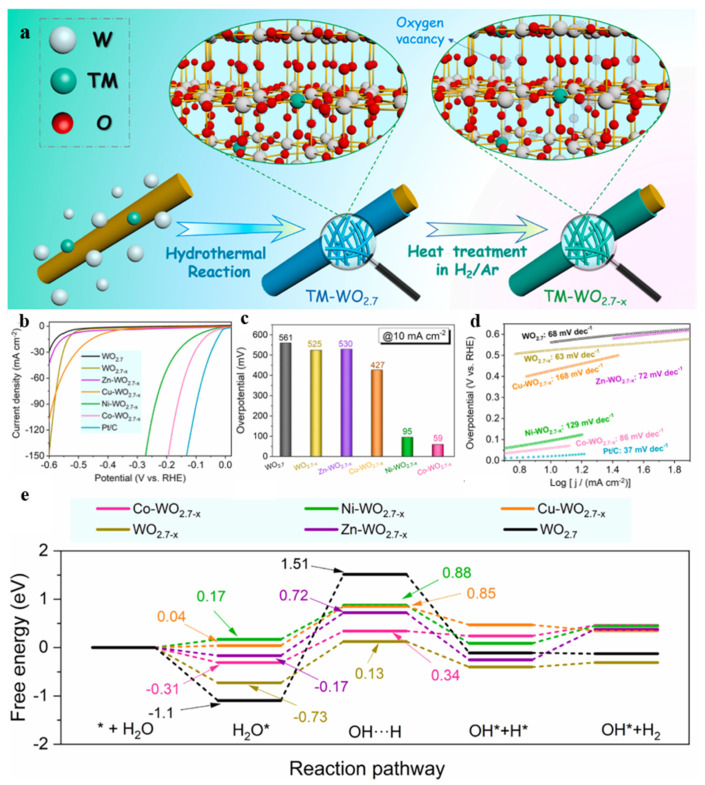
(**a**) A schematic illustration of TM-WO_2.7−x_ preparation. (**b**) LSV curves of as-prepared samples. (**c**) A comparison of the involved overpotentials of different catalysts at 10 mA·cm^−2^. (**d**) The Tafel slopes of all samples measured in a 1 M KOH solution. (**e**) The Gibbs free energy changes of the alkaline HER process on different samples [59].

Cationic doping can also regulate the interface structure, surface chemical state, and band gap; optimize the adsorption energies of reaction intermediates; and thus improve the HER of WO_3_. A summary of studies on metal-doped WO*_3_* catalysts for the HER is presented in Table 2. Liu et al. [96] synthesized self-supported Co-doped WO_3_ on Cu foam, and the overpotentials were 117, 105, and 149 mV at 10 mA·cm^−2^ at the pH values of 0, 7, and 14, respectively. The ΔG_H_ of the W sites on a (200) crystal plane of pure WO_3_ was −0.78 eV, while the ΔG_H_ of the Co sites on a Co-doped WO_3_ surface was 0.44 eV, which was much closer to 0 than that of the W sites in bare WO_3_. These results demonstrated that the improved HER activity could be ascribed to the optimization of the adsorption energy of H* species and electric conductivity caused by Co doping. Deng and their coworkers [56] prepared manganese (Mn)- or vanadium (V)-doped WO_3_ nanoparticles using the hydrothermal method, and the Mn or V impurity atoms replacing the W atom could tune the electronic nature of the WO_3_. Furthermore, the introduction of Mn and V could reduce the free energy of hydrogen adsorption to −0.11 and 0.08 eV, respectively, at O_b_ sites. Therefore, the Mn- and V-doped WO_3_ showed significantly superior HER activity compared to the pure WO_3_, with overpotentials of 97 mV and 38 mV at 10 mA·cm^−2^ and Tafel slopes of 68 and 41 mV·dec^−1^, respectively. Huang et al. [59] regulated the surface electronic structure and morphology of WO_2.7−x_ by doping different metals (Cu, Co, Ni, and Zn), introducing oxygen vacancies, and donating as TM-WO_2.7−x_, and a schematic illustration of the preparation process of TM-WO_2.7−x_ is shown in Figure 4a. Alkaline HER measurements (Figure 4b–d) confirmed that introducing metal dopants could improve the catalytic performance of WO_2.7_. The as-prepared Co-WO_2.7−x_ displayed much better HER activity than Ni-WO_2.7−x_, Cu-WO_2.7−x_, and Zn-WO_2.7−x_, with an overpotential of only 59 mV at 10 mA·cm^−2^ and a Tafel slope of 86 mV·dec^−1^. According to the calculations for the alkaline HER process with different dopants, the Co-WO_2.7−x_ displayed the lowest energy barrier (0.65 eV) for the rate-determining step, namely the water dissociation step. The experimental data were consistent with the results for the calculated charge density distribution, confirming that the surface properties and intrinsic catalytic activity of the catalysts could be adjusted by doping with metal atoms.

#### 3.3.3. Interstitial Atoms

**Figure 5:**
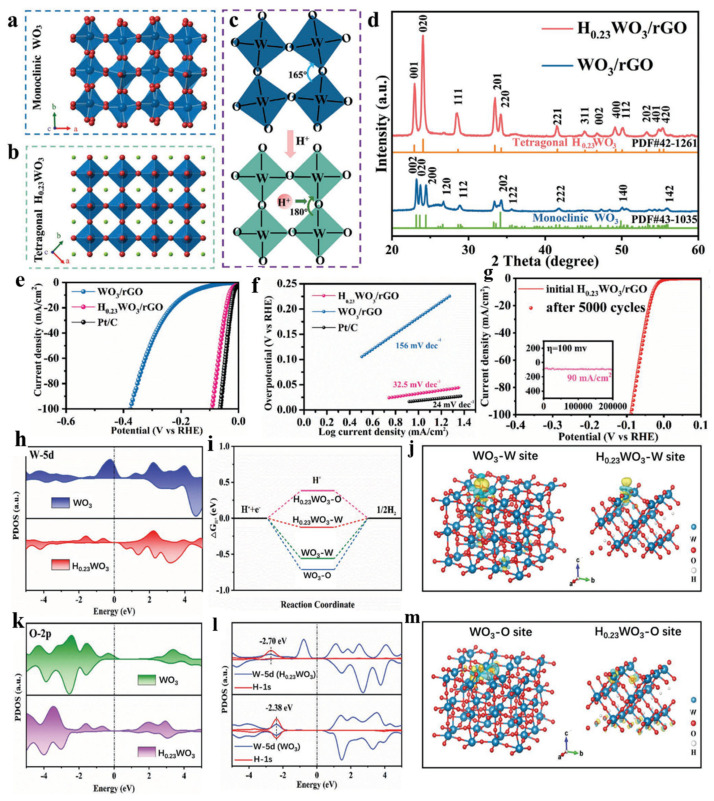
Crystalline structure diagrams of (**a**) monoclinic WO_3_ and (**b**) tetragonal H_0.23_WO_3_. (**c**) The transformation of the crystal structure from monoclinic WO_3_ to tetragonal H_0.23_WO_3_ caused by a hydrogen atom occupying an interstitial site. (**d**) XRD spectra. (**e**) The LSV curves of the samples. (**f**) Tafel slopes. (**g**) The LSV curves of the initial H_0.23_WO_3_/rGO composite and the composite after 5000 cycles. The inset shows a test of H_0.23_WO_3_/rGO stability performed for 200,000 s at 100 mV. All electrocatalytic HER performance tests were performed in 0.5 M H_2_SO_4_. (**h**,**k**) The DOS results for W 5d orbitals and O 2p orbitals on WO_3_ (002) and H_0.23_WO_3_ (020) surfaces. (**i**) Calculated free energy diagrams for the surface W and O sites on WO_3_ (002) and H_0.23_WO_3_ (020). (**l**) PDOS results for the 5d orbitals of surface W atoms on WO_3_ (002) and H_0.23_WO_3_ (020) surfaces after H* adsorption. (**j**,**m**) The variation in charge density at the active sites following hydrogen adsorption on the surface of the catalyst [102].

Interstitial atoms are also an important kind of defect, and a new interstitial atom defect model is proposed to optimize the intrinsic catalytic activity of WO_3_ via modulation of the surface hydrogen adsorption energy. When small hydrogen atoms enter the interstitial lattice position of WO_3_, the strong interaction between interstitial hydrogen and lattice oxygen can significantly reduce the electron density of the d orbital in the active W center, thus weakening the hydrogen adsorption energy of WO_3_ and finally improving the HER performance [102]. Yang and their coworkers annealed ammonium tungstate in the presence of graphite oxide under N_2_, and hydrogen atoms were successfully inserted into the interstitial sites of tungsten oxide (denoted as H_0.23_WO_3_). WO_3_ can form a variety of crystal structures according to the rotation direction and tilt angle of the WO_6_ octahedron. m-WO_3_ presented a perovskite-like structure (Figure 5a) when a hydrogen atom occupied an interstitial position in an m-WO_3_ twisted lattice that was surrounded by near-regular WO_6_ octahedrons, and the angle of the W-O-W bonds changed from 165° to 180°. The rotation direction of the WO_6_ octahedron changed after proton intercalation, which caused the crystalline phase to transform from monoclinic to tetragonal (Figure 5b,c). The crystal phases of as-synthesized catalysts were confirmed by XRD (Figure 5d). The H_0.23_WO_3_/rGO electrocatalyst displayed superior activity, with a low overpotential of 33 mV at 10 mA·cm^−2^ and a Tafel slope of 32.5 mV·dec^−1^ in acidic media (Figure 5e,f). In addition, the H_0.23_WO_3_/rGO catalyst showed amazing stability, and it could work continuously for 200,000 s with no attenuation at a high hydrogen output. The PDOS before (Figure 5h,k) and after (Figure 5l) adsorption of H*, calculated free energy diagrams of the HER (Figure 5i), and the charge density difference (Figure 5j,m) indicated that interstitial H atoms interacted strongly with lattice oxygen in H_0.23_WO_3_, reducing the electron density of the d-orbital W sites on the surface. Therefore, a decrement in the electron density at the active sites of H_0.23_WO_3_ led to a lower hydrogen adsorption energy and weaker W-H bonds compared with WO_3_, which promoted rapid desorption of the surface H* intermediates. Therefore, the H_0.23_WO_3_ catalyst exhibited enhanced kinetic properties and superior catalytic performance in the hydrogen evolution reaction. It could be seen that the surface electronic structure was adjusted and the hydrogen adsorption energy was weakened by inserting hydrogen atoms into the interstitial positions of WO_3_, thereby effectively enhancing its catalytic activity.

The above findings confirm that introducing various defects, such as anion vacancies, cation vacancies, and interstitial atoms, can effectively enhance the electrocatalytic hydrogen evolution properties of WO_3_ nanostructures. However, issues related to the stability of defective WO_3_ during operation in a hydrogen atmosphere remain. For instance, the V-doped WO_3_ prepared by Chandrasekaran et al. exhibited approximately 17.3% current degradation after operating at a constant potential of −100 mV for 14,000 s (less than 4 h) [56]. Therefore, improving the stability of electrocatalysts, particularly at high current densities, represents a critical challenge for future research to meet industrial production requirements.

### 3.4. Constructing Heterostructure

**Figure 6:**
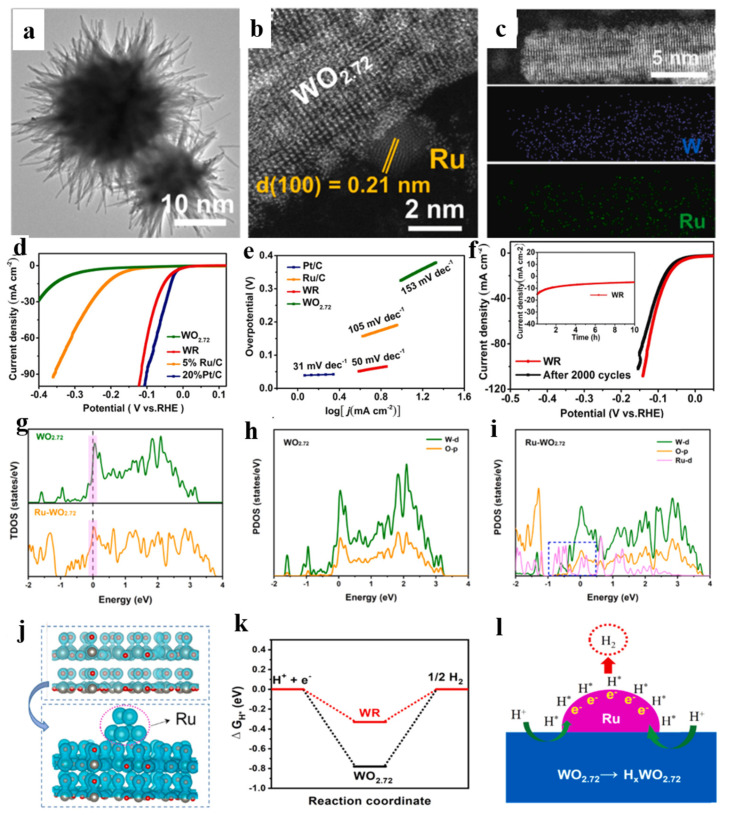
(**a**) TEM, (**b**) HRTEM, (**c**) high-angle annular dark-field scanning transmission electron microscopy, and elemental mapping images of WR. (**d**) LSV curves. (**e**) The Tafel slopes of different samples. (**f**) The LSV curves of the initial WR catalyst and the catalyst after 2000 cycles. The inset shows the chronoamperometry curve of WR at an overpotential of 50 mV. (**g**) The calculated DOS results for WO_2.72_ and WR. (**h**) The PDOS results for WO_2.72_. (**i**) The PDOS results for WR. (**j**) The calculated charge density differences of all samples. (**k**) A free energy diagram of the (010) planes in WO_2.72_ and WR. (**l**) A schematic diagram of the HER mechanism in WR [108].

Constructing heterostructures enables regulation of properties such as electrical conductivity, chemical stability, and hydrophilia through integration with different materials. Furthermore, interface engineering significantly optimizes the hydrogen adsorption free energy and charge transfer kinetics by regulating the surface atomic/electronic structures, the distribution of active sites, and the interfacial reaction environments in electrocatalysts [25,26]. Especially, the Mott–Schottky interface, a metal–semiconductor interface, can effectively adjust the electronic structure at the interface to form a built-in electric field, enhancing the electron transfer capacity, creating nucleophilic/electrophilic regions, and optimizing the adsorption free energy of reaction intermediates [103,104]. Some Mott–Schottky interfaces have been constructed for optimizing HER performance, such as Ni/W_5_N_4_ [105], Ni/CeO_2_ [104], Co/CoP [106], and so on. Chen and their coworkers [107] constructed crystalline cobalt nanoparticles on amorphous tungsten oxide (Co/a-WO_x_). They exhibited excellent HER activity with an overpotential of 36.3 mV at 10 mA·cm^−2^ and a small Tafel slope of 53.9 mV·dec^−1^. Peng et al. [108] prepared a Ru-WO_2.72_ heterostructure (denoted as WR) by adding a minimal number of Ru species to a WO_2.72_ support. It exhibited a spherical structure characterized by numerous radial nanowires (Figure 6a). Ru species measuring about 2 nm were tightly attached to the surfaces of the WO_2.72_ nanowires (Figure 6b,c). The WR required an overpotential of about 40 mV to reach a current density of 10 mA·cm^−2^, and the Tafel slope was 50 mV·dec^−1^ (Figure 6d,e). A chronoamperometry test of the WR electrocatalyst was performed at 10 mA·cm^−2^ and remained stable (Figure 6f). DFT calculations were performed to investigate the electronic and transfer properties of the WR heterostructure. The analysis of charge density differences clearly illustrated a pronounced redistribution of charges following the incorporation of Ru (Figure 6j), which could significantly boost the internal electron concentration of the WR composite, thereby improving the adsorption of H* and the HER properties. The DOS distributions of WR and WO_2.72_ were compared, and the increased electron states at the Fermi level were attributed to the addition of Ru (Figure 6g). A comparison of the partial DOS between WO_2.72_ and WR suggested that the improved DOS near the Fermi level in WR was primarily caused by Ru d orbitals, revealing that the Ru facilitated d-electron donation near the Fermi level (Figure 6h,i). Furthermore, the calculations indicated that hydrogen adsorption had a ΔG_H_ value for WR (−0.33 eV) closer to zero compared to WO_2.72_ (−0.78 eV), which gave rise to much better H* adsorption/desorption on WR (Figure 6k). Based on the results of the experimental analysis and DFT calculations, the Mott–Schottky effect and HER mechanism in WR were discussed (Figure 6l). The work function of WO_2.72_ is lower than that of Ru. Thus, free electrons can spontaneously transfer from WO_2.72_ to Ru until the Fermi levels align, creating an interfacial electric field. Therefore, the redistribution of charges in Ru and WO_2.72_ could result in an electron-rich area on the Ru surface, which would enhance its ability to bind with protons. Moreover, WO_3_ can create a hydrogen–tungsten–bronze compound (H_y_WO_3_) in acidic environments by incorporating hydrogen ions, facilitating the transfer of hydrogen from the active site to the WO_3_ support. The HER process was expedited by the Mott–Schottky structure, which facilitated effective regulation of the electron distribution, and the catalyst underwent a phase transition from WO_2.72_ to H_y_WO_2.72_. This finding confirmed that the Mott–Schottky structure of the catalyst can significantly enhance HER performance by optimizing electronic structures and interfacial charge transfer.

**Figure 7:**
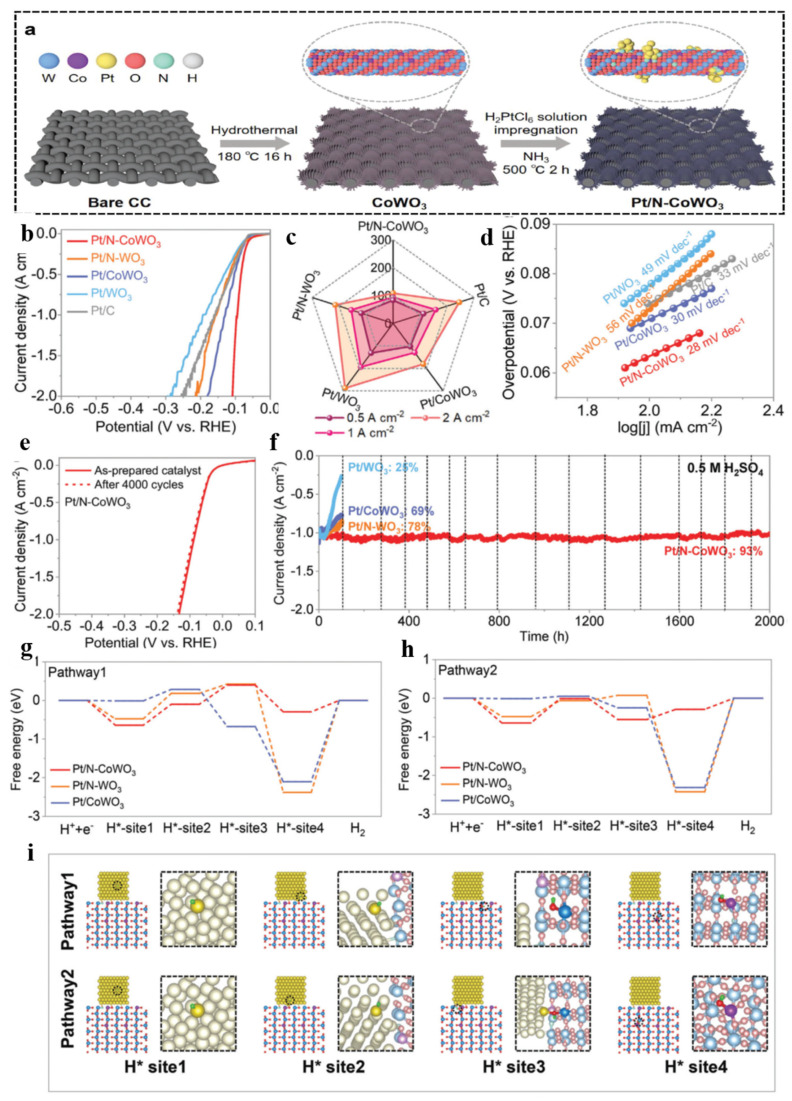
(**a**) A schematic illustration of the preparation process of Pt/N-CoWO_3_. (**b**) The LSV curves of the as-synthesized samples. (**c**) A comparison of the overpotentials at 0.5, 1, and 2 A·cm^−2^. (**d**) Tafel plots. (**e**) The LSV curves of the initial Pt/N-CoWO_3_ catalyst and the catalyst after 4000 cycles. (**f**) The tested stability curves. (**g**,**h**) The DFT calculations of free energy for the HER for different samples. (**i**) The optimized H* adsorption structures at different sites on Pt/N-CoWO_3_ [116].

Currently, Pt-based materials are still the most effective electrocatalysts for the HER in water decomposition. However, the scarcity and high cost of Pt severely restrict its practical application. Therefore, it is of great significance to reduce the contents of Pt in catalysts while maintaining their superior catalytic performance. An effective strategy to achieve this goal is to construct a single-atom catalyst in which Pt (or precious-metal) atoms are isolated and dispersed on the carriers, maximizing the exposure of the active site of Pt, significantly promoting its utilization efficiency or mass activity, and thereby considerably minimizing the amount of Pt in the catalyst. Some electronic metal–support interaction catalysts have attracted attention, such as Pt ACs/CoNCs [109], Pt/RuCeO_x_-PA [110], Pt/N-doped HCS [111], Pt1/OLC [112], and Pt-TiN NTAs [113]. Among them, WO_3_ is considered an excellent carrier because of its superior stability in acidic and neutral media, as well as its reversible and rapid hydrogen insertion [64,114]. Hou et al. first synthesized [115] monolayer WO_3_·H_2_O (ML-WO_3_·H_2_O) using a space-confined strategy, then fixed single Pt atoms (Pt-SA) on monolayer WO_3_ (ML-WO_3_). The Pt-SA/ML-WO_3_ maintained the monolayer structure of ML-WO_3_·H_2_O, with a monolayer ratio of 93% and abundant defects (O and W vacancies). The experimental results showed that Pt-SA/ML-WO_3_ had excellent electrocatalytic performance, with a low overpotential of −22 mV at −10 mA·cm^−2^, a Tafel slope of 27 mV·dec^−1^, a super-high turnover frequency of about 87 H_2_ s^−1^ at −50 mV, and long-term durability. In particular, PT-SA/ML-WO_3_ displayed ultra-high mass activity of 87 A mg_Pt_^−1^ at 50 mV, which was about 160 times higher than that of a commercial catalyst of 20 wt% Pt/C (about 0.54 A mg_Pt_^−1^). According to experimental and density functional theory calculation results, the excellent electrocatalytic HER performance of PT-SA/ML-WO_3_ was attributed to the strong synergistic effect between the single Pt atom and the carrier. Wang et al. [116] prepared Co-doped WO_3_-loading Pt nanoparticles (Pt/N-CoWO_3_) in the presence of ammonia, and a schematic illustration of the preparation process is shown in Figure 7a. As shown by the LSV curves of the as-prepared samples (Figure 7b), the Pt/N-CoWO_3_ catalyst only requires overpotentials of 94 and 108 mV to reach high current density values of 1 and 2 A·cm^−2^, respectively, in 0.5 M H_2_SO_4_, which are lower than those of commercial Pt/C, Pt/WO_3_, Pt/CoWO_3_, and Pt/N-WO_3_ (Figure 7c). The Pt/N-CoWO_3_ catalyst exhibited a much lower Tafel slope of 28 mV·dec^−1^ compared with the other catalysts (Figure 7d). Moreover, the Pt/N-CoWO_3_ exhibited long-term durability for the HER in acidic conditions. The current density remained almost unchanged in the LSV curves after 4000 cycles (Figure 7e). Especially, it could work continuously for more than 2000 h with little attenuation at 1 A·cm^−2^ (Figure 7f), and the morphology and structure of Pt/N-CoWO_3_ had only minor changes after the reaction, showing excellent stability. Two possible H spillover reaction pathways were investigated with Pt as the starting point of the reaction, and free energy diagrams of the HER on different electrocatalysts were generated (Figure 7g,h). For Pt/N-CoWO_3_, interface phases doped with Co, N, and potential CoO sites were studied (Figure 7i). Theoretical calculations indicated that introducing N was more effective for stabilizing Pt, while introducing Co was more effective for the stabilization of W. Meanwhile, the electronic localization function (ELF) confirmed that the interactions between Pt and the substrates in Pt/N-WO_3_ and Pt/N-CoWO_3_ were much stronger than that of Pt/CoWO_3_, indicating that formation of Pt-N/O bonds was the main reason for the stabilization of Pt on the carrier. In addition, Co doping weakened the absorption of O for H at CoO sites, which promoted hydrogen spillover in the WO_3_ phase. Of secondary importance, N can promote the migration of H across the interface. In a word, the synergistic effect of Co and N improved the activity and stability of the HER, which provided guidance for the development of high-performance HER catalysts with multi-functional synergies that run for a long time under acidic conditions.

Of course, WO_3_ can also be combined with other metals or metal compounds (oxides, phosphides, carbides, etc.) to construct highly efficient catalytic systems, and WO_3_-based heterostructure catalysts for the HER are summarized in Table 3. Wang et al. constructed a self-supported porous Ni_17_W_3_/WO_3−x_/MoO_3−x_ heterostructure with excellent HER performance and good stability [117]. Kong and their coworkers synthesized a WS_2_/WO_3−x_ heterostructure enriched with oxygen vacancies, which showed superior HER activity with an overpotential of 120 mV at 10 mA·cm^−2^ and a small Tafel slope of 84.67 mV·dec^−1^ in 0.5 M H_2_SO_4_ [118]. The Ru_2_P/WO_3_@NPC (N and P co-doped carbon) heterostructure was achieved by a simple hydrothermal method using ruthenium and tungsten salts as raw materials [119]. The experimental results displayed that the Ru_2_P/WO_3_@NPC electrocatalyst exhibited outstanding HER activity, with an overpotential of 15 mV at a current density of 10 mA·cm^−2^ and a small Tafel slope of 18 mV·dec^−1^ in a 1.0 M KOH solution, demonstrating superior performance compared with commercial Pt/C and most reported electrocatalysts. Additionally, Ru_2_P/WO_3_@NPC showed robust long-term durability with no significant current decay after a continuous chronoamperometry test. These results, combined with DFT calculations, indicated that the electron density redistribution in Ru_2_P/WO_3_@NPC was facilitated by electron transfer from NPC to Ru_2_P/WO_3_ and from Ru_2_P to WO_3_. The NPC served as a carrier for Ru_2_P/WO_3_, which prevents nanoparticle aggregation, provides electrons to facilitate water dissociation, and could directly promote dissociation of water at the W site and desorption of hydrogen at the Ru site. In a word, efficient interface engineering can accelerate the development of active catalysts for electrocatalytic processes.

### 3.5. Microenvironment Effect

In most cases, traditional strategies to improve catalysts by introducing oxygenophilic active elements, heterostructural regulation, nanoscale limiting, etc., can only gradually or gently adjust the adsorption capacities of electronic states and intermediates, thereby improving catalytic performance. However, the instability of these strategies to eliminate the PH-related dynamics of the HER process prevents them from achieving major breakthroughs in non-acidic electrolytes. In acidic media, the HER performance of catalysts is generally superior due to the higher concentration of H_3_O^+^, which serves as the primary proton source for the reaction. However, in neutral or alkaline conditions, H_2_O is the main proton source; thus, the catalysts are required to overcome a higher energy barrier. Especially, hydroxide ions are nearly absent in neutral electrolytes, and the transfer of intermediates diffusing from the electrode to the electrolyte interface is more complex than in alkaline electrolytes [136]. Consequently, the HER kinetics in neutral/alkaline media are significantly slower than in acidic environments. Therefore, selecting a suitable system to maximize the local acid-like environment through multiple physicochemical effects will provide an alternative strategy for improving electrocatalytic performance in non-acidic electrolytes and designing more efficient electrocatalysts [137].

**Figure 8:**
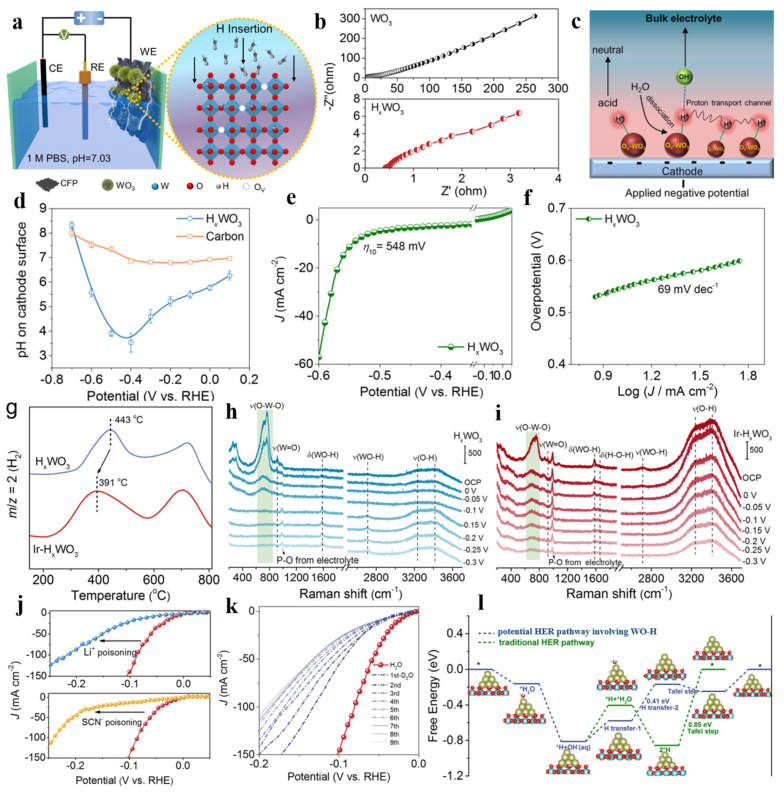
(**a**). Schematic diagram of hydrogen intercalation process of WO_3_ to form H_x_WO_3_. (**b**) EIS Nyquist plots. (**c**) Schematic illustration of local ‘acidic-like’ microenvironment on H_x_WO_3_ electrocatalyst. (**d**) PH values on H_x_WO_3_ surfaces with different potentials. (**e**) LSV of H_x_WO_3_. (**f**) Tafel plots. (**g**) TPD-MS thermal desorption profiles for Ir-H_x_WO_3_ and H_x_WO_3_. Operando electrochemical Raman spectra of (**h**) H_x_WO_3_ and (**i**) Ir-H_x_WO_3_. (**j**) LSV curves of Ir-H_x_WO_3_ before and after poisoning using Li^+^ and SCN^−^. (**k**) LSV curves of Ir-H_x_WO_3_ in 1.0 M PBS (H_2_O and D_2_O). (**l**) Free energy diagram of HER process on Ir_10_-H_x_WO_3_ [64].

The kinetics of the HER is closely related to the properties of the electrode materials and the local reaction microenvironment near the catalytic site at the electrolyte–solid interface [138,139]. Pang’s research team [78] utilized this method to significantly augment the catalytic activity of defect-rich NiS_2_/MoS_2_ nanoflake composites (NMS NFs). The interfacial microenvironment of the NMS NFs could optimize the adsorption energies of reaction intermediates and thereby improve HER performance. Zhao et al. [137] created local acid-like reaction microenvironments on an amorphous NiMoB (am-NiMoB) catalyst in both alkaline and neutral electrolytes, significantly enhancing HER activity. Plenty of hydronium ion (H_3_O^+^) intermediates were generated in situ on the catalyst surface during the HER process, which was verified by operando Raman spectroscopy. The am-NiMoB generally achieved excellent performance related to acidic environments, with overpotentials of 38 mV (1.0 M KOH) and 48 mV (1.0 M PBS) at 10 mA·cm^−2^, and the corresponding Tafel slopes were 34.4 and 39.4 mV·dec^−1^, respectively. In addition, the am-NiMoB catalyst displayed remarkable durability, maintaining operation for over 350 h during seawater electrolysis at industrial current densities (500 and 1000 mA·cm^−2^). Wang et al. [64] reported that WO_3_ achieved reversible and rapid hydrogen doping through electrochemical electron–proton co-doping to form H_x_WO_3_ bronzes at reduction potentials. Hydrogen and oxygen atoms combined to form W-OH species with Brønsted acidity, and the charge rearrangement reduced W^6+^ to W^5+^, which enhanced the conductivity of the electrocatalyst (Figure 8a,b). By testing the local pH of the material, it was observed that protonated H_x_WO_3_ could act as a proton sponge and an electron reservoir to create a more ‘acidic-like’ microenvironment in the electrochemical double layer compared with a traditional carbon substrate (Figure 8c,d). However, a pure H_x_WO_3_ support exhibited poor catalytic activity, which was mainly due to the hindered H-H coupling process on its surface (Figure 8e,f). Thus, additional metal sites needed to be introduced. An Ir-H_x_WO_3_ electrocatalyst was prepared by electrodeposition, and ^1^H solid-state nuclear magnetic resonance and temperature-programmed desorption (TPD) (Figure 8g) measurements confirmed that introducing Ir could enhance the fluidity of protons in the H_x_WO_3_ carrier. Further analysis of the electronic structures of different hydrogen species showed that the interfacial hydrogen species had more electronic states near the Fermi level, indicating that Ir metal could activate the interfacial hydrogen species, enabling them to participate in the HER process. The operando electrochemical Raman spectra of the neutral HER behavior on the H_x_WO_3_ (Figure 8h) and Ir-H_x_WO_3_ surfaces (Figure 8i), as well as poisoning (Figure 8j) and kinetic isotope effect (KIE) (Figure 8k) experiments, showed the synergistic catalytic mechanism of Ir particles and H_x_WO_3_ support. The Ir site in the Ir-H_x_WO_3_ composite had excellent electrocatalytic activity for the Volmer process, which could effectively adsorb H_2_O and further promote its dissociation, thus generating Ir-H_ad_ species at the interface. Ir-H_ad_ spontaneously combined with interfacial activated W_O_-H_ad_ to form H_2_. Meanwhile, the rapid hydrogen transfer on the surface of H_x_WO_3_ could quickly replenish the local hydrogen species consumed at the interface, thus achieving a closed loop for the entire catalytic reaction. Therefore, the superior neutral HER performance of the Ir-H_x_WO_3_ catalyst arises from coherent synergistic catalysis of the Ir metal site and lattice hydrogen species.

## 4. Conclusions and Perspectives

In conclusion, this review mainly summarizes the reasonable design of high-performance electrocatalysts based on WO_3_. Although WO_3_ nanostructures used as electrocatalysts have achieved some research progress in electrocatalytic hydrogen production, there remains a significant gap before practical application, and their HER performance still needs to be further optimized. The surface morphology and crystal phase of WO_3_ can be controlled to adjust band gaps and increase the number of active sites. The electronic structure of WO_3_ can be effectively adjusted by introducing oxygen vacancies and/or atom doping. Stimulating the synergistic effect of multiple phases is used to reduce the reaction barrier of water decomposition and promote adsorption and desorption of protons. In this case, the catalyst has a high electron transport capacity and a large active surface area, which can provide enough active sites and appropriate adsorption strength. It thereby achieves 1 + 1 > 2 in performance. As a result of these efforts, WO_3_ electrocatalysts have been significantly developed. However, the discussion primarily focuses on the HER activity of WO_3_-based nanostructures in alkaline and acidic electrolytes, whereas reports on their HER performance in neutral electrolytes and seawater are relatively scarce. Additionally, most reported WO_3_-based electrocatalysts only exhibit satisfactory catalytic activity at low current densities (≤50 mA cm^−2^) for durations of tens of hours. There is a conspicuous absence of reports on long-term stability (thousands of hours), particularly regarding sustained performance at industrial-grade current densities. These limitations fall short of the requirements for an industrial alkaline electrolyzer.

So far, many achievements have been made in water electrolysis for sustainable hydrogen production, but there is still a long way to go before it is commercially available. Although many challenges remain, there will be many opportunities for researchers to develop this field in the future.

Firstly, the research on the reaction mechanism is not in-depth. Studying the electrocatalytic mechanism is crucial for both deeply understanding the reaction processes and providing theoretical guidance for the design of new materials. Currently, the electrocatalytic mechanisms of most catalysts, particularly composite catalysts, are still not deeply studied. To address this, it is essential to employ theoretical simulations and in situ or operando characterization techniques to support and guide research into catalytic mechanisms. This approach truly integrates experimental design with theoretical analysis, offering theoretical support for the development of efficient electrocatalytic materials.

Secondly, it is necessary to establish standardized measurements. Standardization of testing methods and a unified evaluation system are essential for accurately assessing the performance of electrocatalysts. Various factors, including catalyst loading, electrode preparation methods, and electrolyte composition, can significantly influence catalytic activity. To facilitate fair comparisons and optimization of electrocatalysts, researchers can supply comprehensive data, including parameters like the Tafel slope, exchange current density, catalyst loading amount, electrode surface area, Faraday efficiency, and stability. Establishing standardized protocols ensures that evaluations of electrocatalytic performance are consistent and reliable, aiding in the selection and optimization of superior existing catalysts and the development of new materials. In addition, if necessary, they could also be notarized by third-party testing organizations.

Thirdly, although several strategies have been reported to prepare efficient HER catalysts based on precious metals, the synthesis processes are complex and expensive, resulting in high costs for the final products. The yields and quality of catalysts are not sufficient to satisfy industrial and commercial demand. Significant progress has been made in the development of highly efficient non-precious transition metal-based HER electrocatalysts, but only a few electrocatalysts have performance comparable to Pt-based catalysts, including their catalytic activity, durability, and stability in a wide pH range. Some research groups use precious-metal doping or disperse Pt-based precious metals onto non-precious-metal carriers, which can reduce the loading of precious metals as much as possible while maintaining the high activity and high stability of precious metals, such as the preparation of atomic-level catalysts or the synthesis of catalysts with a hollow or core–shell structure. The operating costs of the precious-metal catalyst are reduced, and the utilization efficiency of the precious-metal catalyst is improved. Boosting the catalytic performance of existing transition metal-based catalysts and exploring new catalysts will be key research objectives in the years to come.

## Figures and Tables

**Table 1:** A summary of pure WO_3_ catalysts with different morphologies for the HER.

Catalysts	Electrolyte	Overpotential (mV)	Current Density (mA·cm^−2^)	Tafel Slope (mV·dec^−1^)	References
WO_3_ nanoparticles	1 M H_2_SO_4_	800	45		[66]
WO_3_ nanoplates	1 M H_2_SO_4_	200	17.58	122	[63]
m-WO_3_ nanorods	1 M H_2_SO_4_	200	23.86	113	[63]
WO_3_ nanoparticles	0.5 M H_2_SO_4_	200	0.72	29	[72]
WO_3_ nanoparticles	1 M KOH	800	0.11	114	[72]
WO_3_ nanorods	1 M H_2_SO_4_	800	23	188	[70]
bulk WO_3_	1 M H_2_SO_4_	800	15	213	[70]
WO_3_ nanoplates	0.5 M H_2_SO_4_	106	10	78	[73]
WO_3_ nanorods	0.5 M H_2_SO_4_	83	10	48	[73]
WO_3_ nanowires	1 M H_2_SO_4_	100	38.4	116	[71]
WO_3_ nanorods	0.5 M H_2_SO_4_	152	10	96	[74]
WO_3_ nanorods	1 M KOH	201	10	105	[74]
WO_3_ nanoplates	0.5 M H_2_SO_4_	73	10	39.5	[75]
WO_3_ nanoplates	distilled water	331	1	51.59	[75]

**Table 2:** A summary of metal-doped WO_3−x_ catalysts for the HER.

Catalysts	Electrolyte	Overpotential (mV)	Current Density (mA·cm^−2^)	Tafel Slope (mV·dec^−1^)	References
V-WO_3_	0.5 M H_2_SO_4_	38	10	41	[56]
Mn-WO_3_	0.5 M H_2_SO_4_	97	10	68	[56]
Mo-W_18_O_49_	0.5 M H_2_-saturated H_2_SO_4_	45	10	54	[97]
Pd-W_18_O_49_	0.5 M H_2_SO_4_	137	10	35	[98]
Ni-W_18_O_49_	1 M KOH	240/350	10/100	92	[99]
1 at% Mo-W_18_O_49_	0.5 M H_2_SO_4_	262/462	10/50	49	[100]
Co-WO_2.7−*x*_	1 M KOH	59	10	86	[59]
Ni-WO_2.7−*x*_	1 M KOH	95	10	129	[59]
Zn--WO_2.7−*x*_	1 M KOH	530	10	72	[59]
Ni-WO*x*	1 M KOH	40.51/137.04	10/100	40	[101]
Ni-WO*x*	1 M KOH seawater	45.69/125.81	10/100	46	[101]

**Table 3:** A summary of catalysts with WO_3-x_-based heterostructures for the HER.

Catalysts	Electrolyte	Overpotential (mV)	Current Density (mA·cm^−2^)	Tafel Slope (mV·dec^−1^)	References
W/WO_2_	1.0 M KOH	35	10	34	[58]
Ni_2_P/WO_2.83_	1.0 M KOH	22.8/254.5	10/1000	53	[61]
WO_3−x_@CdS_1−x_	1.0 M KOH	191	10	61.9	[69]
Co/a-WO_x_	1.0 M KOH	36.3/55.1	10/20	53.9	[107]
Ru-WO_2.72_	0.5 M H_2_SO_4_	40	10	50	[108]
Pt-SA/ML-WO_3_	N_2_-saturated 0.5 M H_2_SO_4_	22	10	27	[115]
Pt/N-CoWO_3_	N_2_-saturated 0.5 M H_2_SO_4_	83/94/108	500/1000/2000	28	[116]
Ni_17_W_3_/WO_3−x_/MoO_3−x_	1.0 M KOH	16	10	34.9	[117]
Ni_17_W_3_/WO_3−x_/MoO_3−x_	0.5 M H_2_SO_4_	14	10	32.6	[117]
Ni_17_W_3_/WO_3−x_/MoO_3−x_	1.0 M PBS	42	10	73.9	[117]
WS_2_/WO_3−x_	0.5 M H_2_SO_4_	120	10	84.67	[118]
WS_2_/WO_3−x_	1.0 M KOH	151	10	97.29	[118]
Ru_2_P/WO_3_@NPC	1.0 M KOH	15	10	18	[119]
WC/WO_3−x_	0.5 M H_2_SO_4_	107	10	59.3	[120]
WC/WO_3−x_	1.0 M KOH	123	10	72.4	[120]
Ar/H_2_-treated WO_3_/C@CoO/NF	1.0 M KOH	55	10	115	[121]
Pt/def-WO_3_@CFC	0.5 M H_2_SO_4_	42	10	61	[122]
WO_3_/Ni_3_S_2_	1.0 M KOH	249	100	45.06	[123]
Ni_2_P-WO_3_/CC	1.0 M KOH	105	10	64.2	[124]
Ni_2_P-WO_3_/CC	0.5 M H_2_SO_4_	107	10	55.9	[124]
Ru-WO_3−x_	1.0 M PBS	19	10	41	[125]
PtCu/WO_3_@CF	0.5 M H_2_SO_4_	41	10	45.90	[126]
Rh-WO_3_	0.5 M H_2_SO_4_	48	10	31	[127]
Rh-WO_3_	1.0 M KOH	116	10	73	[127]
Rh-WO_3_	0.5 M NaCl/1.0 M KOH	98	10	84	[127]
FeCu–BTC/WO_3_–WC	1.0 M KOH	99/220/286	10/50/100	73.2	[128]
Ni/WO_3_	1.0 M NaOH	163	100		[129]
Pt/WO_3_-600	0.5 M H_2_SO_4_	8/26	10/100	35	[130]
WO_x_@C/C	0.5 M H_2_SO_4_	36	60	19.17	[131]
Ru SNC/W_18_O_49_ NWs	0.5 M H_2_SO_4_	21	10	35	[132]
Pt_2_W/WO_3_/RGO	0.5 M H_2_SO_4_	394	500		[133]
Co-WS_2_/WO_3_	0.5 M H_2_SO_4_	321	10	108	[134]
Co-WS_2_/WO_3_	0.5 M KOH	337	10	136	[134]
WO_3_/MWCNT	1.0 M KOH	200	10	70	[135]

## Data Availability

No data was used for the research described in this article.

## Abbreviations

The following abbreviations are used in this manuscript:

HER	hydrogen evolution reaction
WO_3_	tungsten oxide
LSV	linear sweep voltammetry
PDOS	projected density of state
DFT	density functional theory
TMOs	transition metal oxides
OER	oxygen evolution reaction
1D	one-dimensional
3D	three-dimensional
V_O_	oxygen vacancies
CBM	conduction band minimum
